# Origins of the long-range exciton diffusion in perovskite nanocrystal films: photon recycling vs exciton hopping

**DOI:** 10.1038/s41377-020-00443-z

**Published:** 2021-01-01

**Authors:** David Giovanni, Marcello Righetto, Qiannan Zhang, Jia Wei Melvin Lim, Sankaran Ramesh, Tze Chien Sum

**Affiliations:** 1 Division of Physics and Applied Physics, School of Physical and Mathematical Sciences, Nanyang Technological University (NTU), 21 Nanyang Link, Singapore, 637371 Singapore; 2 Energy Research Institute @NTU (ERI@N), Interdisciplinary Graduate School, Nanyang Technological University, 50 Nanyang Avenue, S2-B3a-01, Singapore, 639798 Singapore

**Keywords:** Electronics, photonics and device physics, Micro-optics

## Abstract

The outstanding optoelectronic performance of lead halide perovskites lies in their exceptional carrier diffusion properties. As the perovskite material dimensionality is reduced to exploit the quantum confinement effects, the disruption to the perovskite lattice, often with insulating organic ligands, raises new questions on the charge diffusion properties. Herein, we report direct imaging of >1 μm exciton diffusion lengths in CH_3_NH_3_PbBr_3_ perovskite nanocrystal (PNC) films. Surprisingly, the resulting exciton mobilities in these PNC films can reach 10 ± 2 cm^2^ V^−1^ s^−1^, which is counterintuitively several times higher than the carrier mobility in 3D perovskite films. We show that this ultralong exciton diffusion originates from both efficient inter-NC exciton hopping (via Förster energy transfer) and the photon recycling process with a smaller yet significant contribution. Importantly, our study not only sheds new light on the highly debated origins of the excellent exciton diffusion in PNC films but also highlights the potential of PNCs for optoelectronic applications.

## Exciton hopping: Energy travels far in perovskite nanocrystal films

Excitons quasi-particles in methylammonium lead bromide perovskite nanocrystal films can travel over a surprisingly long distance. Tze Chien Sum and colleagues at Singapore’s Nanyang Technological University uncovered that the long diffusion lengths of more than one micrometer are largely due to excitons hopping from one nanocrystal to another. They are also caused, albeit to a lesser degree, by photon recycling, in which absorbed photons are re-emitted within the material, generating more charge carriers. The team found that perovskite nanocrystals connected by octylamine ligands showed the longest exciton hopping range compared to those connected by hexylamine or oleylamine ligands. The findings demonstrate the potential of lead halide perovskite nanocrystal films for fabricating smaller, faster, and more energy-efficient electronic devices. They also improve the basic understanding of the mechanisms behind long-range energy transport in these films.

## Introduction

The remarkable long and balanced carrier diffusion properties of lead halide perovskites (LHPs) underpin their unprecedented optoelectronic device performance. In 3D (bulk) LHP systems, balanced carrier diffusion lengths exceeding 1 µm have been reported^1,2^, with signatures of ballistic transport in early dynamics^3,4^. Similarly, robust transport properties have recently been demonstrated in quasi-2D LHP systems, enabled by long-range exciton diffusion^5^ and ultrafast spin-preserving exciton transport^6^ over a range of hundreds of nm. Therefore, understanding and optimizing the robust transport properties of LHPs hold the key to their successful application in devices.

Recently, low-dimensional LHPs [e.g., nanocrystals (NCs), quantum dots (QDs), nanorods, and nanowires] have attracted much interest with the promise of further boosting the LHP-based device performance. For instance, the power conversion efficiencies of photovoltaic devices could be improved by carefully tailoring multiple exciton generation (MEG)^7,8^ and hot-carrier cooling processes^9,10^. Moreover, the external quantum efficiencies of LHP-based light-emitting devices have been greatly enhanced by leveraging quantum confinement, resulting in increased absorption cross-sections and photoluminescence quantum yields (PLQYs). However, preserving the excellent transport properties is the toughest hurdle to overcome with the concomitant dimensionality reduction. Firstly, the nature of the carrier transport is altered, as quantum confinement effects enhance excitonic properties, thereby bringing additional factors into play such as the electronic coupling strength between neighboring nanostructures. Secondly, additional sources of static and dynamic disorder are inadvertently introduced due to the size distribution, ligand interdigitation, site vacancies, and generally disordered energy landscape.

Interparticle excitonic interactions in II–VI semiconductor QD films and superlattices have been studied since the first report by Kagan et al.^11,12^. Despite extensive studies and tailoring of the microscopic mechanism of energy transfer (encompassing downhill Förster mediated transfer in an inhomogeneous ensemble), less attention is paid to the exciton spatial propagation properties in QD films. In 2014, Tisdale et al.^13^ reported elegant direct measurement of diffusion lengths in II–VI core–shell QD films based on PL imaging. Unfortunately, small diffusion lengths (~20–30 nm) were reported for CdSe/CdZnS QDs, where the disordered energy landscapes induce subdiffusive exciton transport. Hitherto, these factors have severely constrained the realization of hyper-structured confined-yet-electronically coupled films, which could significantly advance QD-based PVs, display/LED technologies, and lasing fields.

Amid such impasses, lead halide perovskite NCs (PNCs) recently emerged as promising candidates to achieve long-range transport in quantum-confined nanostructures owing to their intrinsically defect-tolerant electronic structure^14–16^ and weakly size-dependent properties^17^. Here, we report the direct measurement of exceptional long-range energy transport of >1 μm in methylammonium lead bromide (MAPbBr_3_) PNC films measured with a direct PL imaging method, surpassing previous reports on other nanostructures. We demonstrate tuning of the exciton mobility in our PNC films up to *D* = 10 ± 2 cm^2^ V^−1^ s^−1^ by simple modification of the organic ligands. Using a phenomenological model, we elucidate the quantitative contributions of inter-NC exciton hopping (EH) and photon recycling (PR) processes to the ultralong exciton diffusion length. Our results not only demonstrate the unprecedented micron-scale diffusion length of excitons in PNCs but also deepen the understanding of the fundamental mechanisms underpinning long-range energy transport in PNC films.

## Results

### Imaging of exciton diffusion in PNC films

**Fig. 1:**
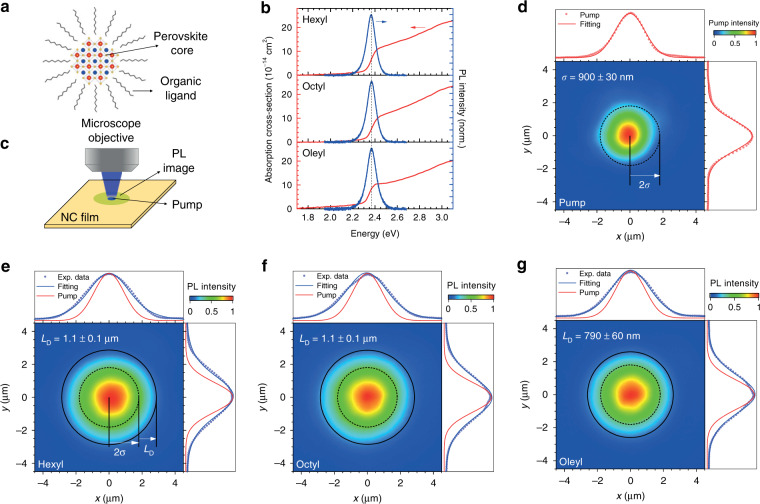
Linear absorption and PL in PNC samples. (**a**) Illustration of our PNC samples. (**b**) Absorption and PL spectra of colloidal MAPbBr_3_ PNCs with different ligands in toluene solution. (**c**) Illustration of the exciton diffusion imaging. (**d**) Image of the TEM_00_ pump excitation spot produced by a 473 nm CW laser, with ***σ*** = 900 **±** 30 nm (FWHM = 2.1 **±** 0.1 μm). The pump profile was fitted with a 2D Gaussian function. (**e**–**g**) PL images of the PNC films with different ligands, whose profiles were fitted with Eq. (2). The x- and y- cross-sections of the PL (blue) and pump (red) are shown for comparison. The dotted line in (**b**) corresponds to the maximum PL wavelength. The dotted and solid lines in the (d–g) contour color plots correspond to circles with radii of 2***σ*** and 2***σ*** + ***L***_**D**_, respectively

MAPbBr_3_ PNCs were synthesized with different organic ligands (Fig. 1a) by slightly modifying a previously reported ligand-assisted reprecipitation method (LARP)^18^. Briefly, PNCs were synthesized by direct precipitation of perovskite precursors in a mixed ligand/benzyl alcohol/toluene phase. The length of the amine ligands was modified while the precursor to ligand molar ratio was kept constant to ensure a consistent synthetic procedure and achieve similar sizes of the PNCs. In this study, we used oleic acid and three different amine ligands: hexylamine, octylamine, and oleylamine (hereafter abbreviated as hexyl, octyl, and oleyl, respectively). Figure 1b presents the absorption and photoluminescence (PL) spectra of the various purified colloidal PNCs. Excitonic absorption resonances peak at approximately 2.40 eV (517 nm) and can be more clearly identified by the minima in the 2^nd^ derivative of the absorption spectra [Supplementary Information, Fig. S1]. Minor differences between samples occur due to the difference in the solubility of the ligands in the antisolvent phase, yielding slightly different growth kinetics^19^. The samples exhibit a strong PL peak at approximately 2.36 eV (526 nm) with minimal Stokes shift. The size distribution from the TEM micrograph (Fig. S2) confirms PNCs with average diameters distributed between 5.7 and 7.7 nm, as shown in Table 1. Notably, the narrow FWHM of ~91 meV (~20 nm), despite the broad size distribution of the PNCs, is in agreement with the well-known weak size dependency^17^. Given the reported exciton Bohr radius of MAPbBr_3_ of ~2 nm^20,21^ and the bulk emission energy *ħω*_bulk_ of ~ 2.30 eV (~540 nm)^22^, our PNCs can be considered weakly confined excitonic systems.

Based on their defect tolerance^14–16^ and weak size dependency^17^, we envisage the possibility of long-range exciton diffusion in PNC films. Comparatively, forerunner studies on II-VI QD films determined that exciton diffusion/transport in such quantum confined systems is largely inhibited by traps, either on the single dot scale, where the surface exhibits midgap energy levels acting as carrier traps, or on the ensemble scale, where the size dependence of energy levels yields larger dots (i.e., lower energy sites) within the ensemble that act as exciton traps^13^. On the other hand, the defect-tolerant properties and size-insensitive electronic structures of PNCs can be leveraged; these properties largely limit the impact of electronic disorder on exciton transport. Moreover, the high PLQY and small Stokes shift in PNCs also promote efficient inter-NC energy transfer (ET) processes, both radiative ET (i.e., PR) and nonradiative ET [e.g., Förster resonance energy transfer (FRET)]. Thanks to these properties, PNCs stem as ideal candidates to achieve long diffusion lengths in quantum-confined systems.

To test our hypothesis, we spatially measured the exciton transport inside PNC films by using a steady-state modification of the PL profile expansion method introduced by Tisdale et al.^13^—Fig. 1c. A continuous-wave (CW) diode laser with a photon energy of 2.62 eV (473 nm) was focused to an ~2 μm spot on the PNC films. The exciton population dynamics *n*(*x*, *y*, *t*) in the steady-state condition can be described by the following differential equation:

1 $$\frac{{\partial n\left( {x,y,t} \right)}}{{\partial t}} = 0 = G\left( {x,y} \right) - \frac{{n\left( {x,y} \right)}}{\tau } + D\nabla ^2n\left( {x,y} \right)$$

The first term on the right-hand side represents the generation rate, which is proportional to the pump intensity profile [i.e., $G\left( {x,y} \right) \propto I_{{\mathrm{pump}}}\left( {x,y} \right)$, which is a Gaussian TEM_00_ mode]; the second term describes the exciton recombination with lifetime *τ*; and lastly, the third term describes the exciton diffusion in two dimensions, with diffusion coefficient *D*. The solution for the differential equation is given by:

2 $$I_{{\mathrm{PL}}}\left( {x,y} \right) \propto {\int \nolimits_{ - \infty }^\infty} {\mathrm{d}}x^{\prime}{\int \nolimits_{ - \infty }^\infty} {\mathrm{d}}y^{\prime}\exp \left( { - \frac{{x^{\prime 2} + y^{\prime 2}}}{{2\sigma ^2}}} \right)K_0\left( {\frac{{\sqrt {\left[ {x^{\prime} - x} \right]^2 + \left[ {y^{\prime} - y} \right]^2} }}{{L_{\mathrm{D}}}}} \right)$$

Details of the derivation can be found in Supplementary Note 1. Here, $I_{{\mathrm{PL}}}$ is the 2D PL image profile observed in our experiment; $K_0\left( x \right)$ is the zeroth-order modified Bessel function of the second kind; and $L_{\mathrm{D}} = \sqrt {D\tau }$ is the exciton diffusion length.

Equation (2) satisfactorily reproduces the 2D PL image profiles of our PNC films, as confirmed by the plots of their x- and y- cross-sections (Fig. 1e–g). The fitting results are reported in Table 1. From the fitting, unprecedented ultralong diffusion lengths ($L_{\mathrm{D}}$) exceeding 1 μm are obtained, which is unusual for such quantum confined systems. For comparison, typical inorganic QD systems (e.g., CdSe QDs) exhibit exciton diffusion lengths of only a few tens of nm^13,23^. The larger values in PNCs correspond to a diffusion coefficient up to 0.27 ± 0.04 cm^2^ s^−1^, which is equivalent to an exciton mobility of $\mu = eD/k_{\mathrm{B}}T$ = 10 ± 2 cm^2 ^V^−1 ^s^−^^1^. Such neutral exciton diffusion is different from charge carrier diffusion in bulk semiconductors. Nevertheless, it is still interesting to note that this exciton mobility is comparable to/larger than the reported typical carrier mobility for bulk 3D perovskite films^24^, i.e., averages of 2.4 ± 1.1 cm^2^ V^−^^1^ s^−1^ and ~8.6 cm^2^ V^−1^ s^−^^1^ for MAPbI_3_ and MAPbBr_3_ thin films, respectively. These results imply that the reduction of the transport properties due to quantum confinement and insulating ligands is fully compensated by the efficient inter-QD ET in PNC films.

**Table 1:** Properties of MAPbBr_3_ PNCs and their films

	Hexyl	Octyl	Oleyl
Size (nm)	6 ± 2	6 ± 2	8 ± 3
PLQY (%)	55 ± 2	65 ± 2	40 ± 2
Exciton energy (eV)	2.40	2.42	2.39
Absorption cross-section at 400 nm (10^−14^ cm^2^)^*^	23 ± 2	23 ± 3	20 ± 2
Effective lifetime (ns)^**^	58 ± 5	42 ± 3	41 ± 4
Diffusion length (μm)	1.1 ± 0.1	1.1 ± 0.1	0.79 ± 0.06
Diffusion coefficient (cm^2 ^s^−1^)	0.20 ± 0.03	0.27 ± 0.04	0.15 ± 0.02
Exciton mobility (cm^2^ V^−1^ s^-1^)	8 ± 1	10 ± 2	5.9 ± 0.8

The size distributions, PLQYs, exciton resonance energies, and other measured parameters of the PNCs are given. Diffusion lengths, diffusion coefficients, and exciton mobilities are given for the corresponding films

^*^Measured by Poisson distribution fitting (Supplementary Note 2)

^**^Measured at a low pump fluence, corresponding to a population of N ~ 0.07 per NC

These excellent exciton transport properties in PNC films could stem from two possible mechanisms. The first is the radiative (or trivial) ET mechanism, i.e., PR, where emitted photons from one NC are reabsorbed by the neighboring NCs. PR has also been reported to enhance not only the transport properties in other types of perovskite systems^25–31^ but also LED performances^32^. The second mechanism is the EH via nonradiative ET between neighboring NCs [e.g., FRET, Dexter energy transfer (DET), or other mechanisms]. Herein, we seek to explicate the quantitative contributions of these two mechanisms in our PNC films.

### Contribution from PR

**Fig. 2:**
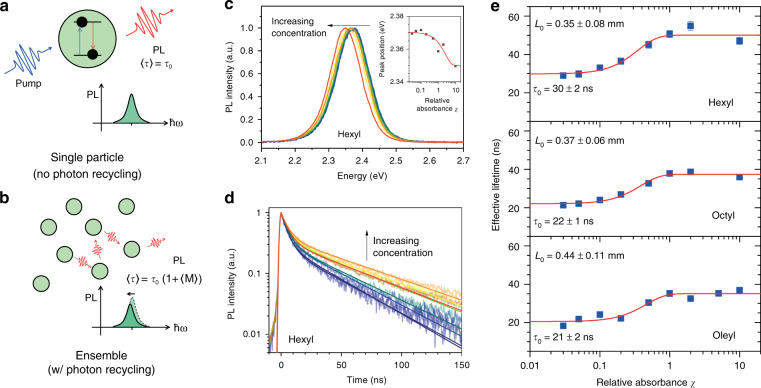
Effect of PR in colloidal PNCs. (**a**, **b**) Illustration of the effect of PR on the PL spectra and lifetimes. (**c**) Redshifting PL spectra and (**d**) increasing PL lifetime of our colloidal PNCs. The inset in fig. (**c**) shows the peak position as a function of concentration, as fitted by the reabsorption model (Supplementary Note 3). (**e**) Effective PL lifetimes of our PNCs with different ligands as a function of the relative colloidal absorbance (χ). The data were fitted with our PR model. The samples were excited with a 3.1 eV pump with a fluence corresponding to ***N*** ~ 0.6 excitons per NC

Figure 2a, b illustrates the PR mechanism and signatures. For a single NC emitter (no PR), the absorption of a photon creates an exciton, which will recombine radiatively after an average time $\langle\tau\rangle = \tau _0$ (Fig. 2a). This photon could then be immediately captured by the time-resolved detection system for lifetime measurement. However, in the case of NC ensembles (with PR), the photon will be reabsorbed and reemitted by the neighboring NCs an average $\langle{M}\rangle$ number of times before leaving the ensemble and being recorded by the time-resolved detection equipment (Fig. 2b). In this case, the detector will measure an increased lifetime of $\langle\tau\rangle = \tau _0(1 + \langle{M}\rangle)$.

Another signature of PR is spectral redshift due to the higher absorption of blue light for any system with PLQY below unity during the reabsorption and reemission processes. To elucidate this process in our PNC system, we performed a time-resolved PL study on the colloidal PNC systems. Colloidal PNCs provide an ideal platform to understand the PR process for several reasons. First, the concentration of NCs per unit volume in the colloidal system ensures sufficient distance between NCs to prevent nonradiative ET processes from occurring. The typical NC concentration obtained from our in situ solution-processed synthesis is ~0.6 μM, corresponding to an average interparticle distance of ~0.14 μm. For comparison, nonradiative ET processes such as FRET or DET could only occur within a distance of at most a few nm^33–35^.

Second, the colloidal system also provides a facile platform for modulating the NC concentration, and hence controlling the PR mean free path (MFP) and the average number of reabsorption-reemission processes *M*. These variables are crucial for quantifying the contribution of PR in PNC films. Indeed, PR signatures were revealed as we modulated the concentration of our colloidal NCs. As the concentration increases, the photon MFP inside the solution decreases, resulting in an increased *M*. Namely, a photon that originates from a given depth inside a cuvette will be recycled (i.e., absorbed and reemitted) a number of times before it can emerge from the solution to be detected. Figure 2c, d shows clear spectral redshifts of the PL central wavelength, together with increasing PL lifetime with increasing colloidal concentration.

To provide a quantitative description of this process, we modeled the PR in a colloidal system photoexcited by a pump traveling along the positive *z*-direction. The colloidal solution is assumed to be situated inside a cuvette with its two interfaces located at *z* = 0 and *z* = *L*_cuv_ (i.e., *L*_cuv_ is the cuvette thickness, 1 mm). A segment of the random movement of a photon in 3-dimensional space inside the colloidal solution can be described by *L*^2^ = 〈*x*^2^〉 + 〈*y*^2^〉 + 〈*z*^2^〉, where *L* is the photon MFP inside the colloidal solution, while 〈*x*^2^〉, 〈*y*^2^〉, and 〈*z*^2^〉 are the square averages of the displacement of the photon in the *x*−, *y*− and *z*-directions, respectively. Given that the photon is emitted in a random direction, the square average of the displacement will be the same for all directions, i.e., 〈*x*^2^〉 = 〈*y*^2^〉 = 〈*z*^2^〉. Hence, the root-mean-square displacement in the *z*-direction for every PR process is given by:

3 $$z_{{\mathrm{RMS}}} = \sqrt {\langle{z^2}\rangle} = \frac{L}{{\sqrt 3 }}$$

Based on random walk theory, a photon originating from a depth *z* inside the cuvette will experience PR on average *M* times before escaping, i.e., $M\left( z \right) = \left( {z/z_{{\mathrm{RMS}}}} \right)^2 = 3z^2/L^2$. The initial exciton distribution *n*(*z*) created by the photoexcitation is described by $n\left( z \right) = n_0\exp \left( { - \sigma _{{\mathrm{pump}}}cz} \right)$, where $\sigma _{{\mathrm{pump}}}$ and *c* are the absorption cross-section at the pump energy and the concentration of the PNCs, respectively. Taking the average of *M* across the initial photon population, we obtain:

4 $$M = \frac{3}{{L^2}}\frac{{\mathop {\int }\nolimits_0^{L_{{\mathrm{cuv}}}} z^2\exp \left( { - \sigma _{{\mathrm{pump}}}cz} \right){\mathrm{d}}z}}{{\mathop {\int }\nolimits_0^{L_{{\mathrm{cuv}}}} \exp \left( { - \sigma _{{\mathrm{pump}}}cz} \right){\mathrm{d}}z}} = 6\frac{{\sigma _{{\mathrm{PL}}}^2}}{{\sigma _{{\mathrm{pump}}}^2}}\left( {1 - \frac{{A\exp \left( { - A} \right)\left( {1 + A/2} \right)}}{{1 - \exp \left( { - A} \right)}}} \right)$$

where $A = \sigma _{{\mathrm{pump}}}cL_{{\mathrm{cuv}}}$ is the absorption of the system; $\sigma _{{\mathrm{PL}}}$ is the sample absorption cross-section at the PL energy; and the definition $L = \left( {\sigma _{{\mathrm{PL}}}c} \right)^{ - 1}$ has been used. Therefore, the apparent lifetime of the system $\langle\tau\rangle$ due to PR, as a function of the relative concentration of the colloidal solution, is given by:

5 $$\langle\tau\rangle = \tau _0\left( {1 + \langle{M}\rangle} \right) = \tau _0\left[ {1 + 6\xi ^2\left( {1 - \frac{{A_0\chi \exp \left( { - A_0\chi } \right)\left( {1 + A_0\chi /2} \right)}}{{1 - \exp \left( { - A_0\chi } \right)}}} \right)} \right]$$

Here, we define $A_0$ as the standard absorbance at a given arbitrary standard concentration $c_0$; $\tau _0$ is the intrinsic lifetime without PR; $\xi \equiv \sigma _{PL}/\sigma _{{\mathrm{pump}}}$ is the ratio of the absorption cross-sections at the PL and pump energies; and $\chi \equiv A/A_0 = c/c_0$ is the relative absorbance (or equivalent relative concentration) of the colloidal solution with respect to the defined standard. The details of the derivation are provided in Supplementary Note 4. Equation (5) was then used to fit the measured PL effective lifetimes (Supplementary Note 5) of our colloidal NCs as a function of $\chi$, with $A_0$, $\xi$, and $\tau _0$ as the three fitting parameters. The results are presented in Fig. 2e, where our model successfully describes the observed lifetime trends. From the fitting, we estimated the photon MFP at the standard concentration ($\lambda _0$) to be in the range of 400–500 μm. Since $\lambda$ is inversely proportional to the colloidal concentration, these values of $\lambda _0$ could be used as a standard parameter to quantify the effect of PR in our systems, as long as their relative concentrations are known (i.e., $\lambda = \lambda _0/\chi$).

A further validation of this model stems from the consistency of our results, verified by other independent experiments. For instance, Fig. 1b shows the linear absorption cross-section ratio to be ~8 × 10^–14^ cm^2^ and ~2 × 10^–13^ cm^2^ around the PL region and at 400 nm, respectively. This agrees with the fitting value of $\xi$ ~ 0.34 obtained for all our samples. Additionally, we estimated the relative concentration ratio of the NCs in films and in solution ($\chi _{{\mathrm{film}}}$) to be ~10^2^ to 10^3^ (ratio of the linear absorption coefficient around 520 nm). Using these values together with the fitting results, our model accurately estimates all the PNC film lifetimes at the given $\chi _{{\mathrm{film}}}$ for all 3 types of ligands, which are in agreement with the time-resolved PL experimental values. The details are provided in Supplementary Note 6. It is also noteworthy that this model is not only applicable to our PNC systems but also could be applied generally to other systems (e.g., colloidal CdSe QDs and Rh6G organic dye solution, Fig. S3).

### Distinguishing the EH and PR contributions in PNC films

**Fig. 3:**
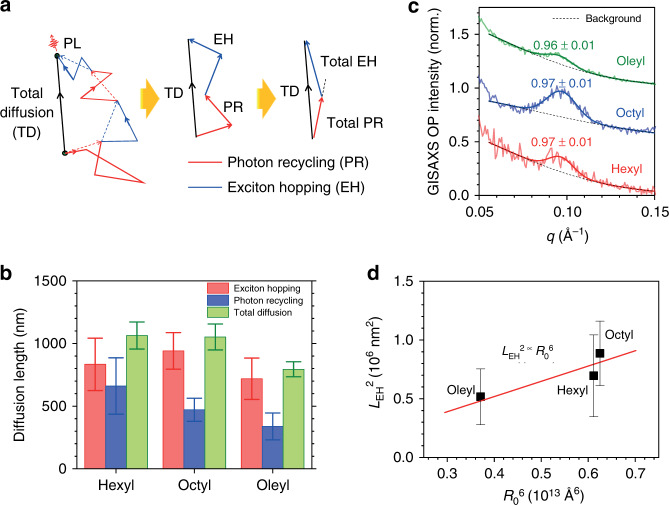
Distinguishing EH and PR in PNC films. (**a**) Illustration of the EH and PR contributions to the total diffusion length (TD), which could be represented as vector addition. (**b**) Estimated quantitative contributions of EH and PR in our PNC films with different ligands. (**c**) Grazing incidence small-angle X-ray scattering (GISAXS) out-of-plane (OP) measurement for our PNC films. The positions of the peaks are labeled. (**d**) Correlation between the EH range (***L***_**EH**_) and FRET range (***R***_0_), fitted with the Smoluchowski-Einstein relation (red line)

We proceed to quantify the individual contributions from PR and EH to the observed ultralong diffusion length in our PNC films. In this case, the relative contributions of the two possible diffusion mechanisms (EH and PR) can be described in the framework of vectorial addition – Fig. 3a. We consider a scenario of exciton diffusion consisting of a series of PR and EH processes with respective total displacement vectors of $\overrightarrow {r_{{\mathrm{PR}}}}$ and $\overrightarrow {r_{{\mathrm{EH}}}}$. The range of the total diffusion ($r_{{\mathrm{TD}}}$) in this scenario can be described by the vectorial addition formula $r_{{\mathrm{TD}}}^2 = \left| {\overrightarrow {r_{{\mathrm{EH}}}} } \right|^2 + \left| {\overrightarrow {r_{{\mathrm{PR}}}} } \right|^2 + \left| {\overrightarrow {r_{{\mathrm{EH}}}} } \right|\left| {\overrightarrow {r_{{\mathrm{PR}}}} } \right|\cos \theta$, where $\theta$ is the angle between the two vectors. The total PR range in the 2D plane here is represented by $L_{{\mathrm{PR}}}$, which is related to the photon MFP in the film ($\lambda _{{\mathrm{film}}}$) by $L_{{\mathrm{PR}}}^2 = 2\langle{M}\rangle\lambda _{{\mathrm{film}}}^2/3$, i.e., in the *x*- and *y*-directions, where $\lambda _{{\mathrm{film}}}$ is given by $\lambda _0$ divided by the concentration ratio between the film and the standard solution (Supplementary Note 6). Averaging over all possible directions, the relation between the total diffusion length ($L_{\mathrm{D}}$), EH range ($L_{{\mathrm{EH}}}$), and PR range ($L_{{\mathrm{PR}}}$) is given by:

6 $$L_{\mathrm{D}}^2 = L_{{\mathrm{EH}}}^2 + L_{{\mathrm{PR}}}^2$$

Based on Eq. (6) and our diffusion length measurement and PR contribution results, we distinguished the quantitative contributions of EH and PR. The result is shown in Fig. 3b. Our results indicate that the EH process dominates diffusion mechanisms in PNC films, with a weaker, albeit considerable, contribution from the PR. Such long-range EH is unprecedented, given the isolated nature of the NCs separated by long insulating ligands.

## Discussion

Interestingly, the octyl-based PNCs show the longest EH range, followed by the hexyl- and oleyl-based systems. To rationalize our findings, we performed grazing incidence small X-ray scattering (GISAXS) measurements to investigate the particle arrangement in the films. Our results reveal out-of-plane stacking in all our PNC films (Fig. 3c), with a characteristic distance of ~65 Å. Assuming that the PNCs are arranged in a hexagonal close-packed (HCP) structure, this value corresponds to a center-to-center interparticle distance of ~79 Å between NCs, similar for all our PNC films. Such invariance is assigned to the oleate ligand present in all samples, which is vital for the stability of the PNCs. This bulky ligand becomes the limiting factor for tuning the interparticle distance within the films. Since our PNC films have similar interparticle distances, we could conclude that the differences in the EH ranges in our PNC films do not originate from their trivial differences in the interparticle distance but rather from their intricate intrinsic photophysical properties.

To delve deeper into the physics of EH in our PNC films, we confirmed the role played by one of the most common mechanisms, i.e., FRET. Within Förster theory, the Förster radius $R_0$ (i.e., the distance at which the transfer efficiency is 50%) can be calculated in Å as^36,37^:

7 $$R_0 = 9.78 \times 10^3\left[ {\kappa ^2n^{ - 4}\eta J} \right]^{1/6}$$

where $\kappa ^2 = 2/3$ is the dipole orientation factor for an isotropic sample; $n = 1.5$ is the medium refractive index (i.e., that of the alkylamine ligands^36^); $\eta$ is the PLQY of our PNC films; and $J$ is the overlap integral between the PL of the donor and absorption of the acceptor (in cm^3^ M^−1^), defined as:

8 $$J = {\int \nolimits_0^\infty} f_{\mathrm{D}}\left( \lambda \right){\it{\epsilon }}_{\mathrm{A}}\left( \lambda \right)\lambda ^4{\mathrm{d}}\lambda$$

Here, $f_D\left( \lambda \right)$ is the normalized PL spectrum of the donor (area = 1); ${\it{\epsilon }}_{\mathrm{A}}\left( \lambda \right)$ is the extinction coefficient of the acceptor (in M^−1^ cm^−1^); and $\lambda$ is the wavelength (in cm). The relation between $\sigma$ and ${\it{\epsilon }}_{\mathrm{A}}$ is presented in Supplementary Note 4. The resulting $R_0$ values for our samples are summarized in Table 2.

**Table 2:** FRET in our MAPbBr_3_ PNCs. The PLQY in the film *η*_*film*_, overlap integral *J*, and FRET range *R*_0_ of the PNCs are given

	Hexyl	Octyl	Oleyl
*η*_*film*_ (%)	51 ± 2	57 ± 2	24 ± 2
*J* (10^-10^ cm^3^ M^-1^)	1.04	0.95	1.34
*R*_0_ (Å)	135	136	124

Thus, our calculation shows remarkable values of $R_0$ in the PNCs, which are one order of magnitude larger than those in typical QD systems (tens of Å)^36,37^. This result implies an efficient FRET process that underpins the unprecedented robust EH in PNC films. To further confirm the role played by the FRET process in the observed EH, we used these *R*_0_ values estimated from independently measured parameters (Table 2) and tested their relationship with the extracted *L*_EH_ using the Smoluchowski-Einstein relation^38^, which dictates the relation of the FRET-driven EH range ($L_{{\mathrm{FRET}}}$) with $R_0$:

9 $$L_{{\mathrm{FRET}}}^{2}={A}\frac{\tau_f}{\tau_0} \frac{R_{0}^{6}}{\tau^4} \propto \frac{{R_{0}^{6}}}{{r^{4}}}$$

where $r$ is the inter-dipole distance; *A* is a constant that accounts for the distribution of molecular separation; *τ*_0_ and *τ*_f_ are the intrinsic and film exciton lifetimes,respectively. Figure 3d shows a correlation between our measured *L*_EH_ and *R*_0_ fitted with the Smoluchowski-Einstein relation. Using our estimated *R*_0_ values and assuming 79 Å inter-particle distance, we obtained a proportionality constant of ~500 [underestimation of *R*_0_ by a factor of ~2.8 times by eq. (7) assuming *A* = 1 and *τ*_0_ = *τ*_f_, and FRET rates (i.e*., τ*_FRET_ ∝ $R_{0}^{6}$) by a factor of ~500]. Such underestimation has also been reported in CdSe QD films, where the calculated *τ*_FRET_ underestimate the actual experimental results by 1–2 orders of magnitude^39^. We believe that a more accurate and possibly quantitative model should not only account for the statistical distribution of the acceptors in the thin film, but also consider the presence of higher multipolar order contributions. However, this is beyond the scope of the current work. Furthermore, there could also be some minor contributions from other processes (e.g., exciton delocalization and Marcus-like charge transfer). However, the presence of a linear correlation between the calculated $R_{0}^{6}$ and $L_{\rm{EH}}^{2}$ confirms that FRET is driving the long-range EH process in PNC films.

In summary, we uncover unprecedented long exciton diffusion lengths exceeding 1 µm in PNC films with magnitudes beyond their quantum sizes. Such long-range neutral exciton diffusion corresponds to mobilities up to 10 ± 2 cm^2^ V^−1cs^ s^−1^, surprisingly outpacing the charge carrier diffusion in the 3D counterparts. Through phenomenological modeling and use of colloidal suspensions as a playground to tune the concentration of the NCs, we distinguish the role of PR and inter-NC EH in PNC films. On a more fundamental level, our method provides a reliable and straightforward way to quantify the exciton transport in nanostructured systems. We discover that the long-range energy transport in PNC films is dominated by FRET. Considering the high PLQY of PNC systems, our findings demonstrate the enormous potential of LHP nanostructures not only for conventional optoelectronic applications (i.e., LEDs) but also for the emerging field of excitonic devices (e.g., exciton transistors^40–42^). Furthermore, the achievement of long-range energy transport is a step towards the implementation of biologically inspired solar cell architectures^43^, where a robust and long-range excitonic transport is key to enable high conversion efficiencies.

## Materials and Methods

### Synthesis of PNCs

Except for methylammonium bromide (MABr) purchased from *Greatcell Solar Material*, all reagents were purchased from *Sigma-Aldrich*. The organic and inorganic salt precursors were stored in a dry N_2_ glovebox, and all other reagents were stored in ambient conditions. The syntheses were carried out in ambient conditions under a fume hood.

MAPbBr_3_ PNCs were synthesized by adapting the protocol previously reported by *Veldhuis* et al. (Ref. ^18^). Briefly, a precursor stock solution was prepared by completely dissolving 117.4 mg of lead bromide (PbBr_2_) and 35.8 mg of MABr in 2 mL of N,N-dimethylformamide (DMF, 99.8% anhydrous). The different NCs were prepared by swift injection of this stock solution (150 μL) into an antisolvent solution under vigorous and homogeneous stirring. For the Hexyl-MAPbBr_3_ PNCs, the antisolvent solution was prepared by adding 1 mL of oleic acid (OAc, 70%), 1 mL of benzyl alcohol (BzOH, 99.8%), and 8 μL of hexylamine to 5 mL of toluene. For the Octyl-MAPbBr_3_ PNCs, the antisolvent solution was prepared by adding 1 mL of oleic acid (OAc, 70%), 1 mL of benzyl alcohol (BzOH, 99.8%), and 20 μL of octylamine to 5 mL of toluene. For the Oleyl-MAPbBr_3_ PNCs, the antisolvent solution was prepared by adding 1.5 mL of oleic acid (OAc, 70%), 1 mL of benzyl alcohol (BzOH, 99.8%), and 35 μL of oleylamine to 5 mL of toluene.

After the injection, a green/yellow suspension was obtained. Purification of the crude material was performed using two centrifugation-redispersion steps at 12000 rpm and 4000 rpm. Subsequently, the precipitated NCs were dispersed in 1.5 mL of anhydrous toluene and stored at 4 °C.

### PL imaging measurement

The PL imaging measurement was performed using a home-built microscope setup, as shown in Figure S6. The pump laser used was a 473 nm continuous wave laser. The microscope objective used was a 50X *Mitutoyo* Plan Apo Infinity Corrected Long WD Objective. The PL image was captured by using a *PCo.edge 4.2* sCMOS camera. An illustration of the setup is shown in Figure S7. Size scaling of the microscope image was performed using a calibration slide. The pump image was collected on a clean glass slide with an additional neutral density filter to prevent saturation. The PL image was collected with an additional *Semrock* 488 nm long-pass filter. No leakage of pump scattering was detected when tested with a non-emitting sample. The pump laser power and camera integration time were kept constant throughout all measurements. The validity of the setup was confirmed with tests on the CdSe/ZnS core-shell QD system (see Supplementary Note 7).

### Linear absorption measurement

The linear absorption measurement was performed with a commercial *Shimadzu UV-3600i* UV-VIS spectrometer.

### Steady-state and time-resolved PL measurement

The steady-state PL and time-resolved PL measurements were performed using our home-built setup, powered by an ~50 fs *Coherent LIBRA*, with a repetition rate of 1 kHz. A 400 nm pump was generated from an 800 nm fundamental beam using a BBO crystal. Short-pass filters were added to block the residual fundamental beams after generation. The emission from the samples was collected with a lens pair and directed either to a monochromator (*Princeton Instrument SP2300i*) and streak camera (*Optronis*) system for time-resolved PL measurement or to a monochromator (*Princeton Instrument SP2300i*) and CCD camera (*Pixis 400b*) system for steady-state PL measurement.

### PLQY measurement

The PLQY measurements were performed using a commercial *Fluorolog 3* spectrofluorometer with an *iHR320* emission monochromator integrated with a *Quanta-Phi 6* integrating sphere.

## Supplementary information

Supplementary Information for Origins of the Long-Range Exciton Diffusion in Perovskite Nanocrystal Films: Photon Recycling vs Exciton Hopping

## Data Availability

The data that support the findings of this study are openly available in DR-NTU (Data) at: https://doi.org/10.21979/N9/6QTW2E. Data are also available from the Corresponding Author upon reasonable request.

## References

[1] Stranks SD (2013). Electron-hole diffusion lengths exceeding 1 micrometer in an organometal trihalide perovskite absorber. Science.

[2] Xing GC (2013). Long-range balanced electron- and hole-transport lengths in organic-inorganic CH_3_NH_3_PbI_3_. Science.

[3] Sung J (2020). Long-range ballistic propagation of carriers in methylammonium lead iodide perovskite thin films. Nat. Phys..

[4] Guo Z (2017). Long-range hot-carrier transport in hybrid perovskites visualized by ultrafast microscopy. Science.

[5] Deng SB (2020). Long-range exciton transport and slow annihilation in two-dimensional hybrid perovskites. Nat. Commun..

[6] Giovanni D (2019). Ultrafast long-range spin-funneling in solution-processed Ruddlesden–Popper halide perovskites. Nat. Commun..

[7] Li MJ (2018). Low threshold and efficient multiple exciton generation in halide perovskite nanocrystals. Nat. Commun..

[8] de Weerd C (2018). Efficient carrier multiplication in CsPbI_3_ perovskite nanocrystals. Nat. Commun..

[9] Chen JS (2019). Cation-dependent hot carrier cooling in halide perovskite nanocrystals. J. Am. Chem. Soc..

[10] Li MJ (2017). Slow cooling and highly efficient extraction of hot carriers in colloidal perovskite nanocrystals. Nat. Commun..

[11] Kagan CR, Murray CB, Bawendi MG (1996). Long-range resonance transfer of electronic excitations in close-packed CdSe quantum-dot solids. Phys. Rev. B.

[12] Kagan CR (1996). Electronic energy transfer in CdSe quantum dot solids. Phys. Rev. Lett..

[13] Akselrod GM (2014). Subdiffusive exciton transport in quantum dot solids. Nano Lett..

[14] Kang J, Wang LW (2017). High defect tolerance in lead Halide perovskite CsPbBr_3_. J. Phys. Chem. Lett..

[15] Akkerman QA (2018). Genesis, challenges and opportunities for colloidal lead halide perovskite nanocrystals. Nat. Mater..

[16] Huang H (2017). Lead halide perovskite nanocrystals in the research spotlight: stability and defect tolerance. ACS Energy Lett..

[17] Kim YH (2017). Highly efficient light-emitting diodes of colloidal metal–halide perovskite nanocrystals beyond quantum size. ACS Nano.

[18] Veldhuis SA (2017). Benzyl alcohol-treated CH_3_NH_3_PbBr_3_ nanocrystals exhibiting high luminescence, stability, and ultralow amplified spontaneous emission thresholds. Nano Letters.

[19] Huang H (2017). Growth mechanism of strongly emitting CH_3_NH_3_PbBr_3_ perovskite nanocrystals with a tunable bandgap. Nat. Commun..

[20] Tanaka K (2003). Comparative study on the excitons in lead-halide-based perovskite-type crystals CH_3_NH_3_PbBr_3_ CH_3_NH_3_PbI_3_. Solid State Commun..

[21] Wang Q (2018). Quantum confinement effect and exciton binding energy of layered perovskite nanoplatelets. AIP Adv..

[22] Zhang ZY (2016). The role of trap-assisted recombination in luminescent properties of organometal halide CH_3_NH_3_PbBr_3_ perovskite films and quantum dots. Sci. Rep..

[23] Lee EMY, Tisdale WA (2015). Determination of exciton diffusion length by transient photoluminescence quenching and its application to quantum dot films. J. Phys. Chem. C.

[24] Herz LM (2017). Charge-carrier mobilities in metal halide perovskites: fundamental mechanisms and limits. ACS Energy Lett..

[25] Pazos-Outón LM (2016). Photon recycling in lead iodide perovskite solar cells. Science.

[26] Brenes R (2019). Benefit from photon recycling at the maximum-power point of state-of-the-art perovskite solar cells. Phys. Rev. Appl..

[27] Motti SG (2019). Heterogeneous photon recycling and charge diffusion enhance charge transport in quasi-2D lead-halide perovskite films. Nano Lett..

[28] Gan ZX (2019). The dominant energy transport pathway in halide perovskites: photon recycling or carrier diffusion?. Adv. Energy Mater..

[29] Wang YP (2016). Photon transport in one-dimensional incommensurately epitaxial CsPbX_3_ arrays. Nano Lett..

[30] Dursun I (2018). Efficient photon recycling and radiation trapping in cesium lead halide perovskite waveguides. ACS Energy Lett..

[31] Bowman AR (2020). Quantifying photon recycling in solar cells and light-emitting diodes: absorption and emission are always key. Phys. Rev. Lett..

[32] Cho C (2020). The role of photon recycling in perovskite light-emitting diodes. Nat. Commun..

[33] Turro, N. J. Energy transfer processes. in Photochemical Processes in Polymer Chemistry–2 (ed Smets, G.) (Pergamon, 1977), 405–429.

[34] Andrews DL, Curutchet C, Scholes GD (2011). Resonance energy transfer: beyond the limits. Laser Photonics Rev..

[35] Olaya-Castro A, Scholes GD (2011). Energy transfer from Förster–Dexter theory to quantum coherent light-harvesting. Int. Rev. Phys. Chem..

[36] Righetto M (2018). Engineering interactions in QDs–PCBM blends: a surface chemistry approach. Nanoscale.

[37] Stewart MH (2013). Competition between Förster resonance energy transfer and electron transfer in stoichiometrically assembled semiconductor quantum dot–fullerene conjugates. ACS Nano.

[38] Mikhnenko OV, Blom PWM, Nguyen TQ (2015). Exciton diffusion in organic semiconductors. Energy Environ. Sci..

[39] Jolene Mork A, Weidman MC, Prins F, Tisdale WA (2014). Magnitude of the Förster Radius in Colloidal Quantum Dot Solids. J. Phys. Chem. C.

[40] Unuchek D (2018). Room-temperature electrical control of exciton flux in a van der Waals heterostructure. Nature.

[41] High AA (2007). Exciton optoelectronic transistor. Opt. Lett..

[42] Kuznetsova YY (2010). All-optical excitonic transistor. Opt. Lett..

[43] Brédas JL, Sargent EH, Scholes GD (2017). Photovoltaic concepts inspired by coherence effects in photosynthetic systems. Nat. Mater..

