# Supplementary material for: Origins of the long-range exciton diffusion in perovskite nanocrystal films: photon recycling vs exciton hopping
Source: Light Sci Appl. 2021 Jan 1;10:2. doi: 10.1038/s41377-020-00443-z (PMC7775951; doi:10.1038/s41377-020-00443-z)
Supplement: Supplementary file 1 — Supplementary Information for Origins of the Long-Range Exciton Diffusion in Perovskite Nanocrystal Films: Photon Recycling vs Exciton Hopping [file 41377_2020_443_MOESM1_ESM.docx]

Supplementary Information for

**Origins of the Long-Range Exciton Diffusion in Perovskite Nanocrystal Films: Photon Recycling vs Exciton Hopping**

David Giovanni^1,†^, Marcello Righetto^1,†^, Qiannan Zhang^1^, Jia Wei Melvin Lim^1,2^, Sankaran Ramesh^1,2^, Tze Chien Sum^1,^*

† Authors contributed equally to the work

^1^Division of Physics and Applied Physics, School of Physical and Mathematical Sciences, Nanyang Technological University (NTU), 21 Nanyang Link, Singapore 637371, Singapore.

^2^Energy Research Institute @NTU (ERI@N), Interdisciplinary Graduate School, Nanyang Technological University, 50 Nanyang Avenue, S2-B3a-01, Singapore 639798, Singapore

*Corresponding Author’s Email: [tzechien@ntu.edu.sg](mailto:tzechien@ntu.edu.sg)

Keywords:

Perovskite Nanocrystals, Photon Recycling, Diffusion Length, PL imaging.

# Supplementary Figures


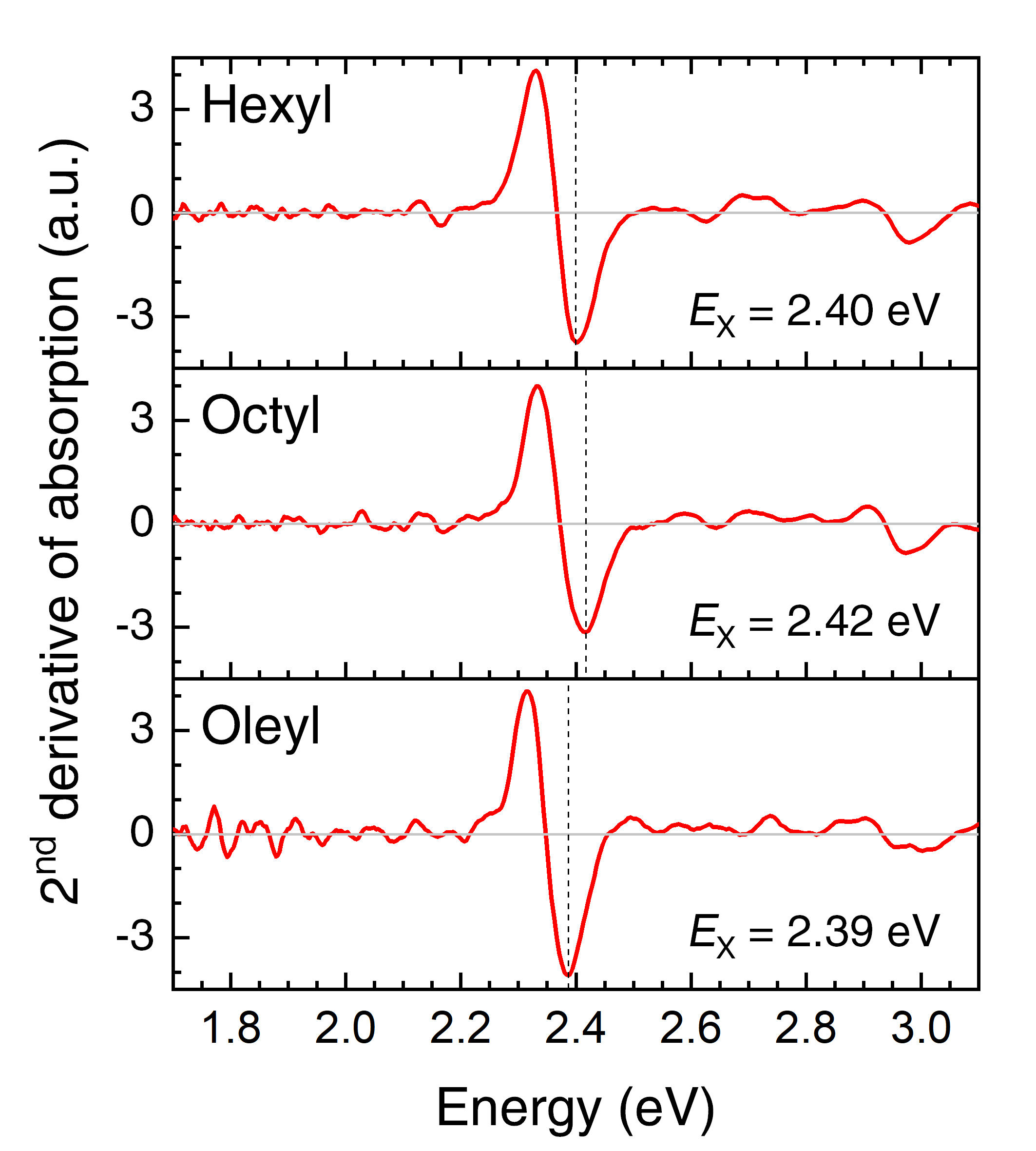


**Figure S1 | The 2^nd^ derivatives of perovskite nanocrystals (PNCs) linear absorption spectra.** The minimum of the 2^nd^ derivatives imply the maximum upward curvature which correspond to the onset of the continuum band absorptions.


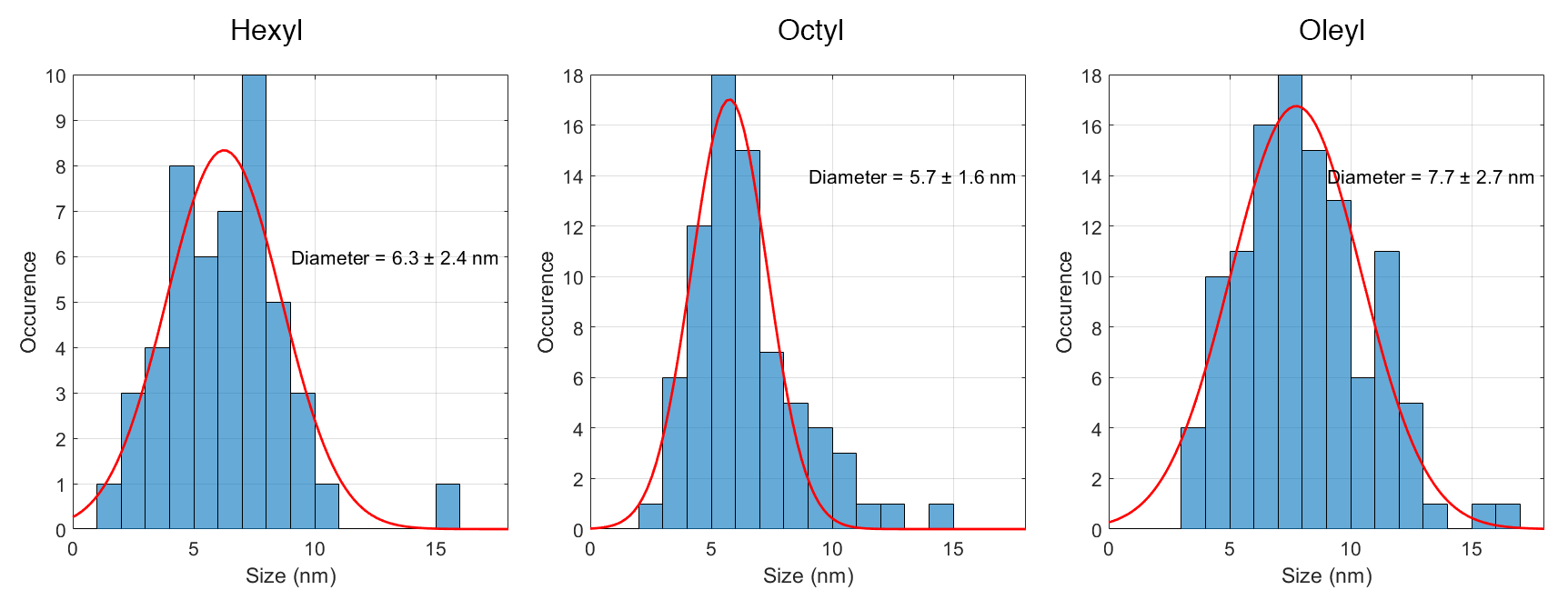


**Figure S2 | Size distribution of our PNCs.** The images show the size distributions of our PNCs for the 3 different ligands obtained from transmission electron microscope (TEM).

**
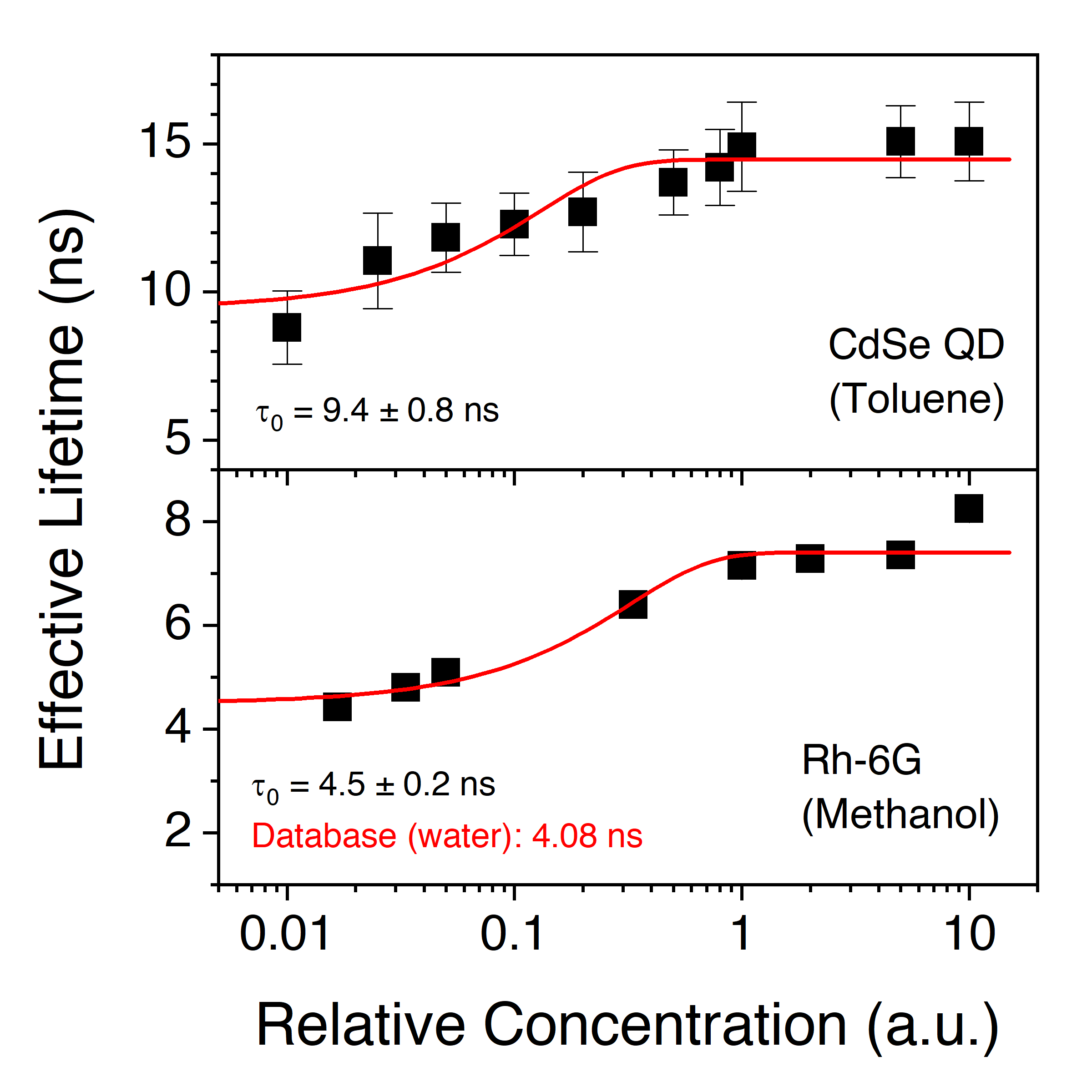
**

**Figure S3 | Lifetime vs concentration for colloidal CdSe QDs and Rhodamine-6G (Rh6G) dye.** The trend is fitted with the photon recycling model [*i.e.*, eq. (5)] in the Main Text. The fitting result of the low concentration lifetime limit for the Rh6G reproduces the standard value obtained from the fluorescence dye online database (<http://www.iss.com/resources/reference/data_tables/FL_LifetimeStandards.html>, accessed 24 Feb 2020).


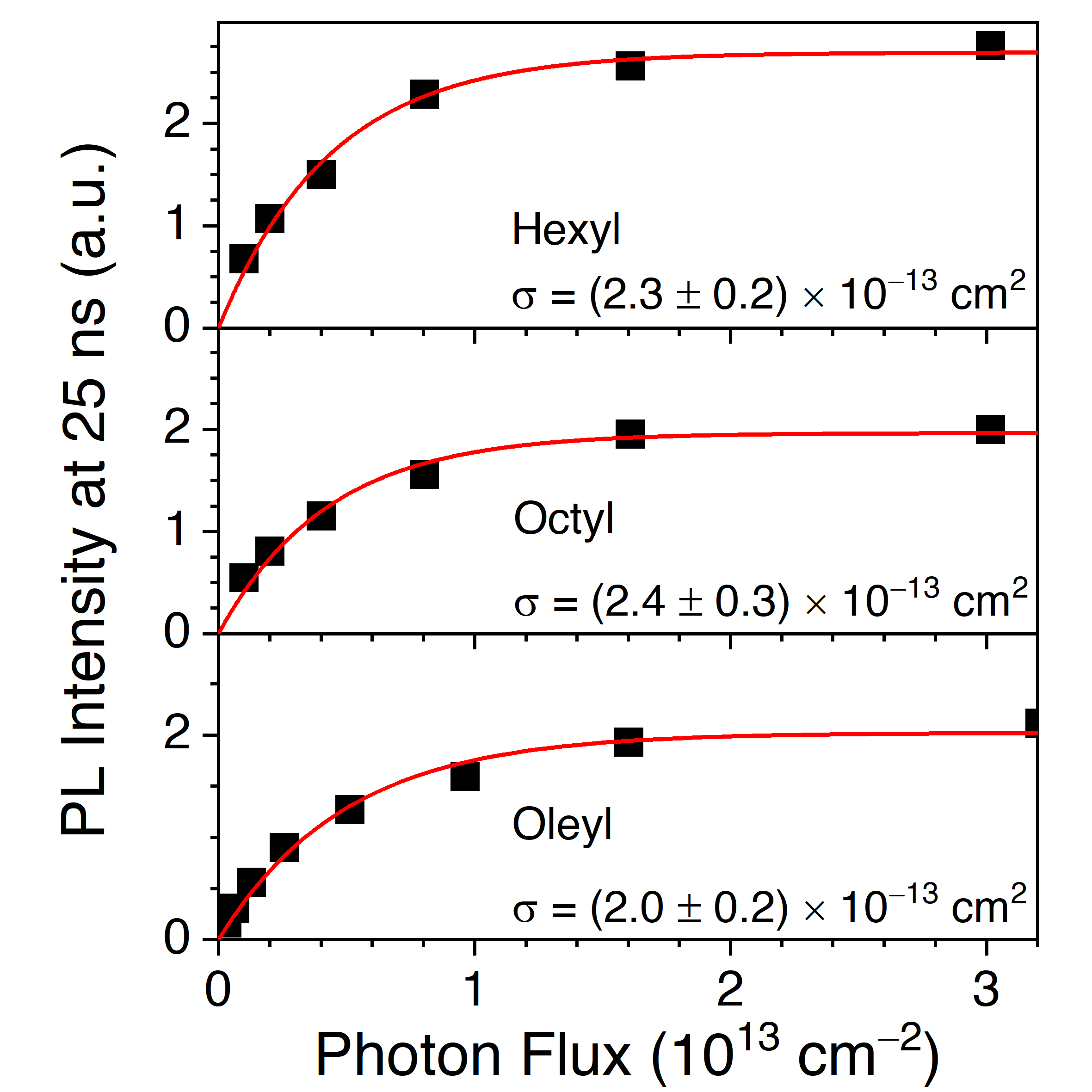


**Figure S4 | Calculation of absorption cross-section by Poisson distribution.** The colloidal PNC samples are photoexcited with 400 nm pulsed laser. The PL intensity is taken at 25 ns.


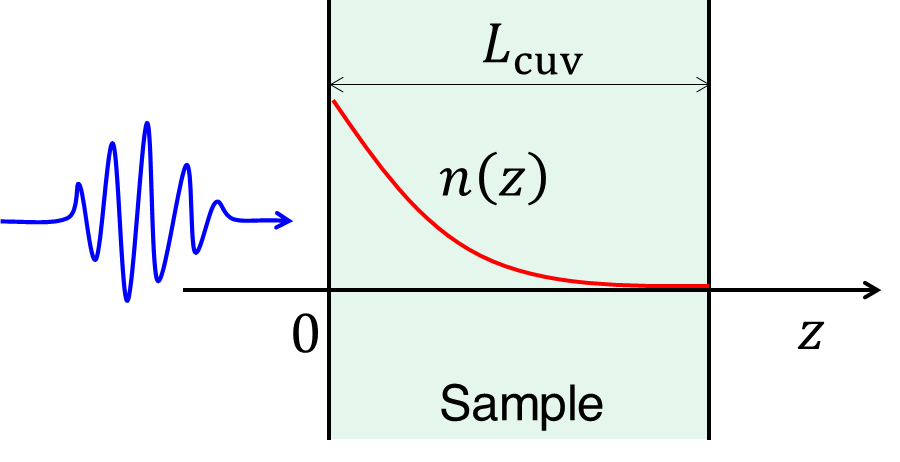


**Figure S5 | Photon recycling (PR) model.** We assume the pump travels in positive *z*-direction, which then creates a distribution of excited states in the sample as function of *z*.


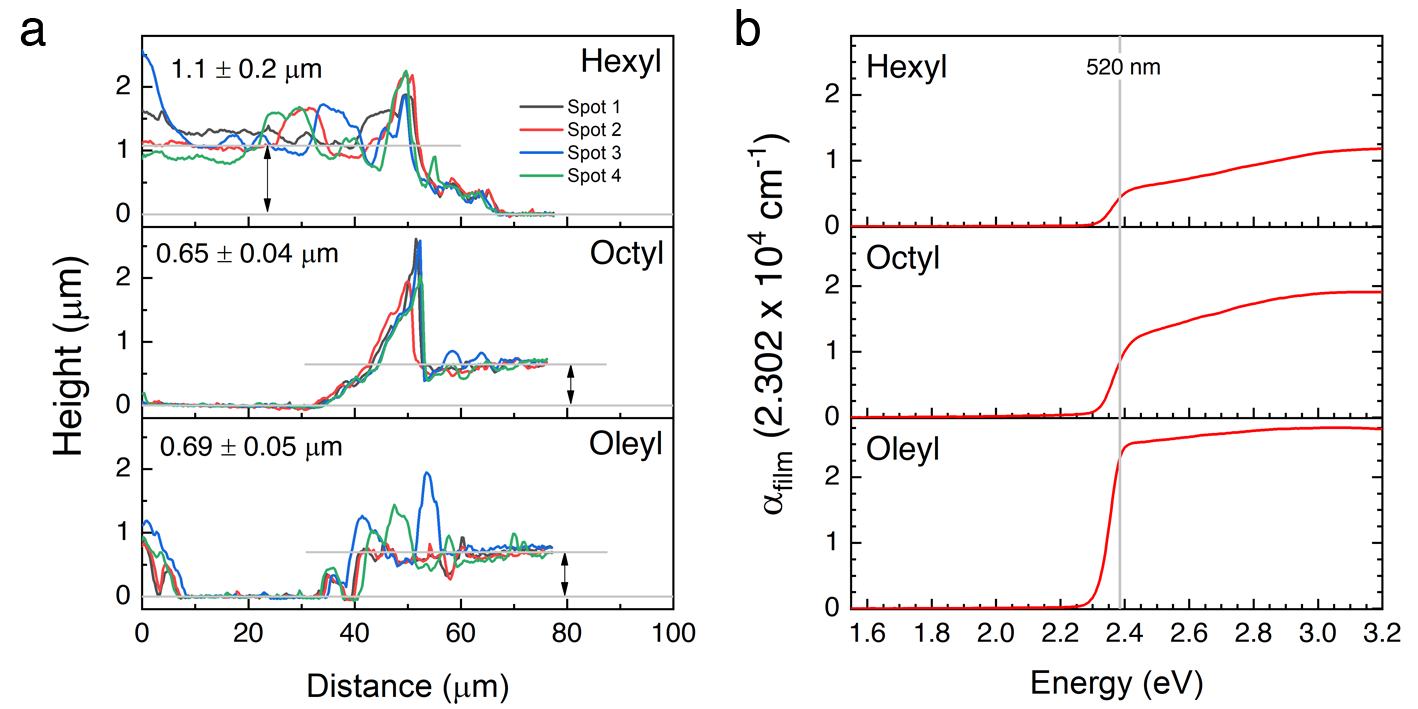


**Figure S6 | Drop-casted perovskite nanocrystal (PNC) films.** (a) The Atomic Force Microscopy (AFM) thickness measurements and (b) the absorption coefficient spectra, of our PNC films for 3 different ligands.

**
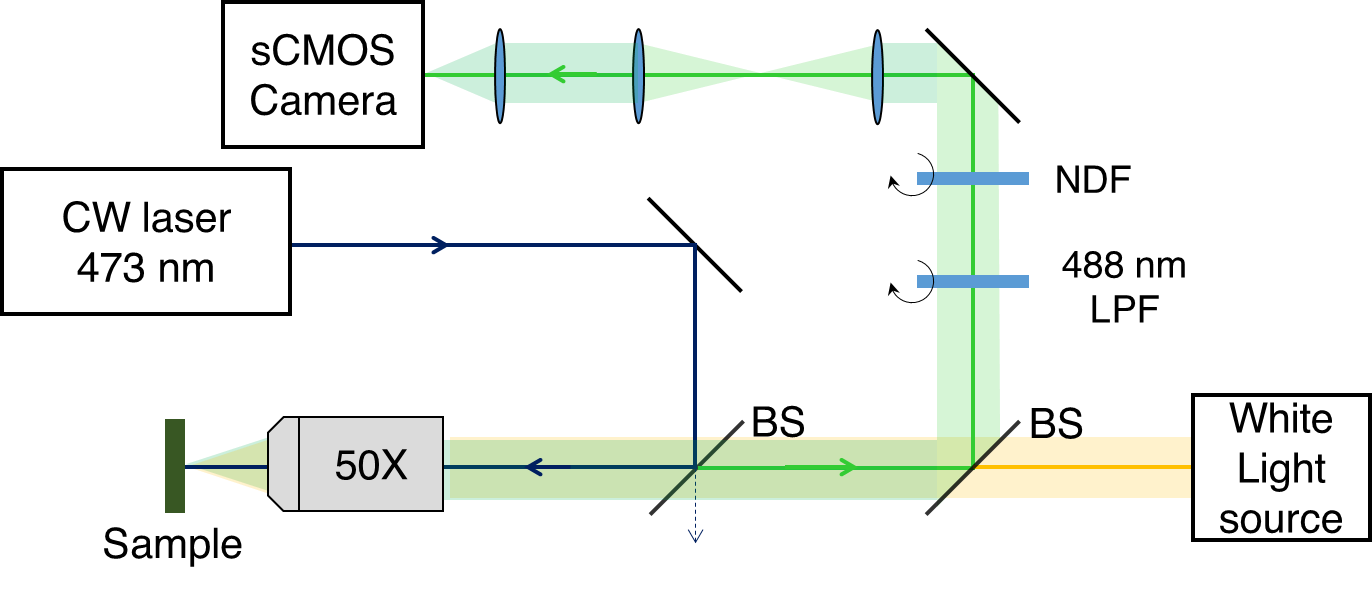
**

**Figure S7 | PL imaging experimental setup.** BS refers to beam-splitter; LPF refers to long-pass filter; NDF refers to natural density filter.


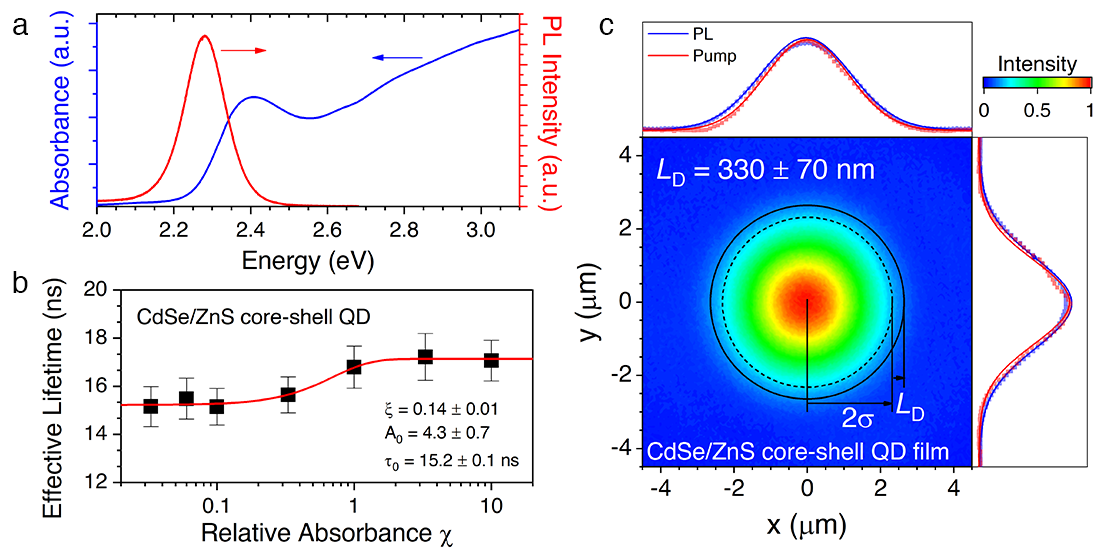


**Figure S8 | Measurement on CdSe/ZnS core-shell QD.** (a) Absorption and PL spectra of the QD solution. (b) Effective lifetime as function of relative concentration/absorbance of the QD solution, fitted with eq. (5) in Main Text. (c) PL imaging measurement on thin QD film, revealing total diffusion length $L_{D}$ = 330 ± 70 nm. The dotted and solid line indicate the radii of $2\sigma$ (of the Gaussian pump) and $2\sigma+L_{D}$, respectively.

# Supplementary Note 1: Derivation of 2D exciton diffusion equation

As described in the Main Text, the steady-state exciton diffusion dynamics can be described by the following differential equation:

|  | $\frac{\partial n\left( x,y,t \right)}{\partial t}=0=G\left( x,y \right)-\frac{n\left( x,y \right)}{\tau}+D\nabla^{2}n\left( x,y \right).$ | (S1) |
| --- | --- | --- |

The $n\left( x,y,t \right)\equiv n\left( x,y \right)$ is the exciton population density; the $G\left( x,y \right)$ represents the exciton generation rate; $\tau$ is the exciton effective lifetime; and $D$ is the diffusion coefficient. Here, the system is assumed to be cylindrically symmetric *i.e.*, $n\left( x,y \right)\equiv n\left( r,\theta\right)\equiv n\left( r \right)$, where $r=\sqrt{x^{2}+y^{2}}$. Consider the following differential equation:

|  | $\delta\left( r \right)-\frac{n\left( r \right)}{\tau}+D\nabla^{2}n\left( r \right)=0$ | (S2) |
| --- | --- | --- |

where $\delta\left( r \right)$ is the Dirac delta function (*i.e.*, point source), and $\nabla^{2}$ is the Laplacian in cylindrical coordinate, which is given by:

|  | $\nabla^{2}n\left( r \right)=\frac{1}{r}\frac{d}{dr}\left( r\frac{d}{dr}n\left( r \right) \right).$ | (S3) |
| --- | --- | --- |

Defining $\psi\left( r \right)=r^{1/2} n\left( r \right)$ and substituting to eq. (S2), the equation can be simplified into:

|  | $\frac{d^{2}}{dr^{2}}\psi\left( r \right)+\left[ \frac{1}{4r^{2}}-\frac{1}{L_{D}^{2}} \right]\psi\left( r \right)=0$ | (S4) |
| --- | --- | --- |

which holds for $r>0$. Here, $L_{D}=\sqrt{D\tau}$ is the exciton diffusion length. The solution of this differential equation is given by:

|  | $\psi\left( r \right)=c_{1}\sqrt{r}J_{0}\left( i\frac{r}{L_{D}} \right)+c_{2}\sqrt{r}Y_{0}\left( -i\frac{r}{L_{D}} \right),$ |  |
| --- | --- | --- |
|  | $n\left( r \right)=c_{1}J_{0}\left( i\frac{r}{L_{D}} \right)+c_{2}Y_{0}\left( -i\frac{r}{L_{D}} \right)$ | (S5) |

where $c_{1}$ and $c_{2}$ are constants; $J_{0}\left( x \right)$ and $Y_{0}\left( x \right)$ are the 0^th^-order Bessel function of the 1^st^ and 2^nd^ kind, respectively. Given the boundary conditions: $n\left( r\to0 \right)=1$ and $n\left( r\to\infty\right)=0$, the solution can be simplified into:

|  | $n\left( r \right)=-iJ_{0}\left( i\frac{r}{L_{D}} \right)-Y_{0}\left( -i\frac{r}{L_{D}} \right)=\frac{2}{\pi}K_{0}\left( \frac{r}{L_{D}} \right).$ | (S6) |
| --- | --- | --- |

Here, $K_{0}\left( x \right)$ is the 0^th^-order modified Bessel function of the second kind. For the case of TEM_00_ pump, the generation rate is given by:

|  | $G\left( x,y \right)=I_{0}\exp\left[ -\frac{x^{2}+y^{2}}{2\sigma^{2}} \right].$ | (S7) |
| --- | --- | --- |

The solution for such generation rate is given by the 2D convolution of the solution for $G\left( x,y \right)=\delta\left( x,y \right)$, *i.e.*, eq. (S6), with eq. (S7); that is:

|  | $n\left( x,y \right)\propto\int_{-\infty}^{\infty} dx^{'}\int_{-\infty}^{\infty} dy^{'}\exp\left[ -\frac{{x^{'}}^{2}+{y^{'}}^{2}}{2\sigma^{2}} \right]K_{0}\left( \frac{\sqrt{\left[ x^{'}-x \right]^{2}+\left[ y^{'}-y \right]^{2}}}{L_{D}} \right).$ | (S8) |
| --- | --- | --- |

# Supplementary Note 2: Determination of linear absorption cross-section

To determine the absorption concentration, we assumed a Poisson distribution for photon absorption in the perovskite nanocrystals PNCs. Assuming that an ensemble of nanocrystals is initially photoexcited by a laser pulse, the probability for a nanocrystal to absorb $k$ number of photons (*i.e.*, to contain $k$ number of exciton) is described by:

|  | $P_{k}=\frac{\left\langle N \right\rangle^{k}}{k!}e^{-\left\langle N \right\rangle},$ | (S9) |
| --- | --- | --- |

where $\left\langle N \right\rangle=\sigma J$ is the initial average number of excitons per nanocrystal in the ensemble. Here, $J$ corresponds to the flux of the pump pulse (in photon/cm^2^); and $\sigma$ is the absorption cross-section of the nanocrystals at pump energy/wavelengths.

At a later time beyond the lifetime of multi-exciton processes (typically > 1 ns), the remaining excitons will mainly recombine via single exciton emission process. The PL intensity could then be assumed to be proportional to the occupation probability of the nanocrystals (*i.e.*, number of nanocrystals that initially contain at least an exciton), that is:

|  | $I_{\mathrm{PL}}\propto1-P_{0}=1-e^{-\sigma J},$ | (S10) |
| --- | --- | --- |

where $P_{0}=e^{-\sigma J}$ is the probability for the nanocrystal not to absorb any photon/contain any exciton. Fitting Eq. (S10) into our time-resolved PL kinetics at 25 ns, we obtained the absorption cross-sections for our PNCs. The result is shown in Figure S4.

# Supplementary Note 3: Modelling of PL redshift due to reabsorption effect

We analyzed the redshifting effect of PL spectra due to reabsorption process as function of sample absorbance. Assuming an emitting sample in a cuvette with thickness $L_{\mathrm{cuv}}$, the apparent PL spectrum $I_{\mathrm{PL}}^{'}\left( \omega\right)$ is given by:

|  | $I_{PL}^{'}\left( \omega\right)=\frac{1}{L_{\mathrm{cuv}}}\int_{0}^{L_{\mathrm{cuv}}} dz I_{\mathrm{PL}}\left( \omega\right)e^{-\varepsilon\left( \omega\right)cz}=\frac{I_{\mathrm{PL}}\left( \omega\right)}{\varepsilon\left( \omega\right)cL_{\mathrm{cuv}}}\left( 1-e^{-\varepsilon\left( \omega\right)cL_{\mathrm{cuv}}} \right),$ | (S28) |
| --- | --- | --- |

where $I_{\mathrm{PL}}\left( \omega\right)$ is the PL spectrum of a single nanocrystal (without reabsorption effect); $c$ is the concentration; and $\varepsilon\left( \omega\right)$ is the extinction coefficient.

Without the loss of generality, we assumed a gaussian single-particle PL with peak position $\omega_{0}$ and broadening $\sigma$:

|  | $I_{\mathrm{PL}}\left( \omega\right)=I_{0}\exp\left( -\frac{\left( \omega-\omega_{0} \right)^{2}}{2\sigma^{2}} \right).$ | (S29) |
| --- | --- | --- |

The peak position of the apparent PL spectrum ($\omega_{R}$) can be described as:

|  | $\left. \frac{d}{d\omega}I_{PL}^{'}\left( \omega\right) \right\vert_{\omega=\omega_{R}}=0.$ | (S30) |
| --- | --- | --- |

Solving these equations, we obtained:

|  | $\omega_{R}=\omega_{0}-\frac{\sigma^{2}}{\varepsilon\left( \omega_{R} \right)}\left. \frac{d\varepsilon\left( \omega\right)}{d\omega} \right\vert_{\omega_{R}}\left( 1-\frac{\varepsilon\left( \omega_{R} \right)cL_{\mathrm{cuv}}e^{-\varepsilon\left( \omega_{R} \right)cL_{\mathrm{cuv}}}}{1-e^{-\varepsilon\left( \omega_{R} \right)cL_{\mathrm{cuv}}}} \right)$ $=\omega_{0}-A\left( 1-\frac{B\chi e^{-B\chi}}{1-e^{-B\chi}} \right).$ | (S31) |
| --- | --- | --- |

Here, $A$ and $B$ are positive constants, which are treated as fitting parameters; and $\chi=\varepsilon\left( \omega_{R} \right)cL_{\mathrm{cuv}}$ is the relative absorbance of the sample at $\omega_{R}$. As expected, reabsorption effect will cause the apparent PL peak to redshift from $\omega_{0}$. This relation indeed could describe our experimental data very well, as shown in the inset of Fig. 2c in the Main Text.

# Supplementary Note 4: Derivation of the photon recycling model

We modelled the photon recycling (PR) process as the brownian motion of a photon inside a colloidal solution. A segment of the random movement of a photon in 3-dimensional space inside the solution is described by $\lambda^{2}=\left\langle x^{2} \right\rangle+\left\langle y^{2} \right\rangle+\left\langle z^{2} \right\rangle$, where $\lambda$ is the photon mean-free-path (MFP); while $\left\langle x^{2} \right\rangle$, $\left\langle y^{2} \right\rangle$, and $\left\langle z^{2} \right\rangle$ are the square average of the displacement of the photon in *x*-, *y*- and *z*-direction, respectively. Since the direction is random, the square average of the displacement will be the same for all directions, *i.e.*, $\left\langle x^{2} \right\rangle=\left\langle y^{2} \right\rangle=\left\langle z^{2} \right\rangle$. Hence, the root-mean-square displacement in *z*-direction for every PR process is given by:

|  | $z_{\mathrm{RMS}}=\sqrt{\left\langle z^{2} \right\rangle}=\frac{\lambda}{\sqrt{3}}.$ | (S11) |
| --- | --- | --- |

We assumed excitation in along positive z-direction which hits the sample with thickness $L_{\mathrm{cuv}}$ (*i.e.*, either a cuvette or a film) at z = 0 (Figure S5). The excited state distribution inside the sample is given by $n\left( z \right)=n_{0}\exp\left( -\sigma_{\mathrm{pump}}cz \right)$, where where $n_{0}$ is the maximum exciton density; $\sigma_{\mathrm{pump}}$ is the pump absorption cross-section; and *c* is the concentration of the NCs. Assuming a backscattering PL detection scheme, based on random walk theory a photon originating from a depth *z* inside the cuvette in average would experience $M$ times of PR process in order to escape:

|  | $M\left( z \right)=\left( \frac{z}{z_{\mathrm{RMS}}} \right)^{2}=3\frac{z^{2}}{\lambda^{2}}.$ | (S12) |
| --- | --- | --- |

Given the initial exciton distribution $n\left( z \right)$ inside the sample, the average number of PR across the sample can be estimated as:

|  | $\left\langle M \right\rangle=\frac{3}{\lambda^{2}}\frac{\int_{0}^{L_{\mathrm{cuv}}} z^{2}\exp\left( -\sigma_{\mathrm{pump}}cz \right) dz}{\int_{0}^{L_{\mathrm{cuv}}} \exp\left( -\sigma_{\mathrm{pump}}cz \right) dz}$ $=\frac{3}{\lambda^{2}} \frac{\frac{2}{\left( \sigma_{\mathrm{pump}}c \right)^{3}}\left[ 1-e^{-A}-Ae^{-A}-\frac{1}{2}A^{2}e^{-A} \right]}{\frac{1}{\sigma_{\mathrm{pump}}c}\left( 1-e^{-A} \right)}$ $=\frac{6}{\sigma_{\mathrm{pump}}^{2}c^{2}\lambda^{2}}\left( 1-\frac{Ae^{-A}\left( 1+A/2 \right)}{1-e^{-A}} \right).$ | (S13) |
| --- | --- | --- |

Here, $A\equiv\sigma_{\mathrm{pump}}cL_{\mathrm{cuv}}$ is the absorbance of the sample at pump wavelength/energy. Hence, the equation can be re-written into:

|  | $\left\langle M \right\rangle=\frac{6}{A^{2}}\frac{L_{\mathrm{cuv}}^{2}}{\lambda^{2}} \left( 1-\frac{Ae^{-A}\left( 1+A/2 \right)}{1-e^{-A}} \right).$ | (S14) |
| --- | --- | --- |

We utilized the mathematical definition of MFP for PR process and re-wrote it as:

|  | $\lambda=\frac{1}{\sigma_{\mathrm{PL}}c}=\frac{L_{\mathrm{cuv}}}{A}\frac{\sigma_{\mathrm{pump}}}{\sigma_{\mathrm{PL}}}=\frac{L_{\mathrm{cuv}}}{A\xi},$ | (S15) |
| --- | --- | --- |

where $\sigma_{\mathrm{PL}}$ is the photon absorption cross-section at PL energy/wavelength; and $\xi\equiv{\sigma_{\mathrm{PL}}}/{\sigma_{\mathrm{pump}}}$. We also defined the absorbance ratio ($\chi$) as the ratio of the actual sample absorbance with respect to an arbitrarily set standard absorbance $A_{0}$:

|  | $\chi\equiv\frac{A}{A_{0}}$ | (S16) |
| --- | --- | --- |

Equation (S14) can therefore be written as:

|  | $\left\langle M \right\rangle=6\xi^{2}\left( 1-\frac{A_{0}\chi e^{-A_{0}\chi}\left( 1+{A_{0}\chi}/2 \right)}{1-e^{-A_{0}\chi}} \right).$ | (S17) |
| --- | --- | --- |

The apparent lifetime of the sample at a given relative absorbance/concentration $\chi$ is therefore given by:

|  | $\left\langle\tau\right\rangle=\tau_{0}\left( 1+\left\langle M \right\rangle\right)=\tau_{0}\left[ 1+6\xi^{2}\left( 1-\frac{A_{0}\chi e^{-A_{0}\chi}\left( 1+{A_{0}\chi}/2 \right)}{1-e^{-A_{0}\chi}} \right) \right],$ | (S18) |
| --- | --- | --- |

where $\tau_{0}$ is the sample intrinsic lifetime without the presence of PR. Eq. (S18) is equivalent to Eq. (5) in the Main Text. This model could satisfactorily describe our experimental results, with $A_{0}$, $\xi$, and $\tau_{0}$ as fitting parameters. In this study, the standard absorbance $A_{0}$ is defined as the absorbance of our PNCs solutions with the as-synthesized concentration ($c_{0}$, typically ~0.3 μM) in a 1-mm cuvette (*i.e.*, $L_{\mathrm{cuv}}$ = 1 mm). Also note that this standard absorbance refers to the absorbance of the sample in the region where the PR occurs (*i.e.*, the overlapping region between absorption and PL spectra, which is ~520 nm). Under the standard condition (*i.e.*, $\chi$ = 1), the standard photon MFP ($\lambda_{0}$) is given by:

|  | $\lambda_{0}=\frac{L_{\mathrm{cuv}}}{A_{0}\xi}.$ | (S19) |
| --- | --- | --- |

It is noteworthy to clarify some of convention that is used in this work. The absorption cross-section ($\sigma$), absorbance ($A$), thickness $L$, and absorption coefficient ($\alpha$) are related to each other by:

|  | $A=\alpha L=\sigma cL.$ | (S20) |
| --- | --- | --- |

Here, $A$ is defined in the base of Euler (instead of the base of 10), with a unit of $\log_{10} \left( e \right)$ OD ≈ 0.434 OD; the unit of $\alpha$ is cm^-1^; the unit of $\sigma$ is cm^2^; and the unit of $c$ is cm^-3^. The extinction coefficient $\epsilon$ (in cm^-1^ M^-1^) is related to the $\sigma$ by:

|  | $\epsilon\left( \mathrm{cm}^{-1} M^{-1} \right)=\sigma\left( \mathrm{cm}^{2} \right)\times N_{A}\times\frac{{10}^{-3}}{\ln\left( 10 \right)},$ | (S21) |
| --- | --- | --- |

where $N_{A}$ = 6.022 × 10^23^ is the Avogadro number.

# Supplementary Note 5: Effective lifetime calculation

It is noteworthy to mention that our PNC samples display a bi-exponential decay behavior (with components and lifetimes of $A_{1,2}$ and $\tau_{1,2}$, respectively), which is a typical characteristic of trapping and de-trapping process. In this case, we are interested in the effective lifetimes of the samples, which are calculated as:

|  | $\tau_{\mathrm{eff}}=\frac{A_{1}\tau_{1}^{2}+A_{2}\tau_{2}^{2}}{A_{1}\tau_{1}+A_{2}\tau_{2}}.$ | (S22) |
| --- | --- | --- |

The details of the fitted lifetimes and their component amplitude are presented in Table S1

**Table S1 | Effective lifetimes of PNC solutions with varying relative absorbance** $\boldsymbol{\chi}$**.** The individual lifetime components are presented

| **Hexyl** | | | | | |
| --- | --- | --- | --- | --- | --- |
| $\boldsymbol{\chi}$ | $\boldsymbol{A}_{\boldsymbol{1}}$ **(%)** | $\boldsymbol{\tau}_{\boldsymbol{1}}$ **(ns)** | $\boldsymbol{A}_{\boldsymbol{2}}$ **(%)** | $\boldsymbol{\tau}_{\boldsymbol{2}}$ **(ns)** | $\boldsymbol{\tau}_{\mathbf{eff}}$ **(ns)** |
| 0.03 | 76.8 ± 0.4 | 4.4 ± 0.1 | 23.2 ± 0.2 | 37.6 ± 0.3 | 28 ± 1 |
| 0.05 | 77.6 ± 0.4 | 4.4 ± 0.1 | 22.4 ± 0.2 | 37.1 ± 0.3 | 28 ± 1 |
| 0.1 | 74.8 ± 0.4 | 4.8 ± 0.1 | 25.2 ± 0.2 | 41.3 ± 0.3 | 32 ± 1 |
| 0.2 | 73.0 ± 0.4 | 5.1 ± 0.1 | 27.0 ± 0.2 | 44.6 ± 0.3 | 35 ± 1 |
| 0.5 | 70.5 ± 0.4 | 5.4 ± 0.1 | 29.5 ± 0.2 | 53.0 ± 0.3 | 44 ± 2 |
| 1 | 70.2 ± 0.4 | 5.7 ± 0.1 | 29.8 ± 0.2 | 61.8 ± 0.4 | 52 ± 2 |
| 2 | 68.4 ± 0.4 | 5.8 ± 0.1 | 31.6 ± 0.2 | 64.8 ± 0.4 | 55 ± 2 |
| 10 | 71.0 ± 0.4 | 5.5 ± 0.1 | 29.0 ± 0.2 | 61.4 ± 0.4 | 51 ± 2 |
| **Octyl** | | | | | |
| 0.03 | 77.0 ± 0.4 | 4.2 ± 0.1 | 23.0 ± 0.3 | 30.3 ± 0.3 | 22 ± 1 |
| 0.05 | 75.8 ± 0.4 | 4.1 ± 0.1 | 24.2 ± 0.3 | 30.3 ± 0.3 | 22 ± 1 |
| 0.1 | 74.2 ± 0.4 | 4.4 ± 0.1 | 25.8 ± 0.2 | 31.8 ± 0.2 | 24 ± 1 |
| 0.2 | 71.8 ± 0.4 | 4.6 ± 0.1 | 28.2 ± 0.2 | 34.8 ± 0.3 | 27 ± 1 |
| 0.5 | 69.3 ± 0.4 | 5.1 ± 0.1 | 30.7 ± 0.2 | 42.3 ± 0.3 | 34 ± 1 |
| 1 | 70.1 ± 0.4 | 5.4 ± 0.1 | 29.9 ± 0.2 | 49.4 ± 0.3 | 40 ± 1 |
| 2 | 68.6 ± 0.4 | 5.5 ± 0.1 | 31.4 ± 0.2 | 50.5 ± 0.3 | 42 ± 2 |
| 10 | 70.9 ± 0.4 | 5.3 ± 0.1 | 29.1 ± 0.2 | 50.3 ± 0.3 | 41 ± 2 |
| **Oleyl** | | | | | |
| 0.03 | 66.1 ± 0.5 | 3.9 ± 0.1 | 33.9 ± 0.4 | 24 ± 2 | 19 ± 1 |
| 0.05 | 68.9 ± 0.4 | 5.6 ± 0.1 | 31.1 ± 0.4 | 28.7 ± 0.2 | 22 ± 1 |
| 0.1 | 67.6 ± 0.4 | 5.9 ± 0.1 | 32.4 ± 0.4 | 32.0 ± 0.3 | 25 ± 1 |
| 0.2 | 67.3 ± 0.4 | 5.4 ± 0.1 | 32.7 ± 0.4 | 29.2 ± 0.2 | 23 ± 1 |
| 0.5 | 68.4 ± 0.3 | 6.8 ± 0.1 | 31.6 ± 0.3 | 41.2 ± 0.3 | 33 ± 1 |
| 1 | 68.9 ± 0.3 | 6.9 ± 0.1 | 31.1 ± 0.2 | 48.4 ± 0.3 | 40 ± 1 |
| 2 | 68.3 ± 0.3 | 6.6 ± 0.1 | 31.7 ± 0.3 | 44.6 ± 0.3 | 37 ± 1 |
| 5 | 66.2 ± 0.3 | 7.2 ± 0.1 | 33.8 ± 0.3 | 47.0 ± 0.3 | 39 ± 1 |
| 10 | 68.4 ± 0.3 | 7.4 ± 0.1 | 31.6 ± 0.2 | 52.1 ± 0.3 | 43 ± 1 |

# Supplementary Note 6: Photon recycling in perovskite nanocrystal films

Based on its mathematical definition, the mean-free-path (MFP) is known to be inversely proportional to the concentration – Eq. (S15). Thus, the photon MFP in our PNC films ($\lambda_{\mathrm{film}}$) can be estimated given value of $L_{0}$ (*i.e.*, MFP at $\chi=1$), and the relative PNC concentration ratio in the film and standard solution ($\chi_{\mathrm{film}}^{'}$):

|  | $\lambda_{\mathrm{film}}=\frac{\lambda_{0}}{\chi_{\mathrm{film}}^{'}}.$ | (S23) |
| --- | --- | --- |

To calculate the value of $\chi_{\mathrm{film}}^{'}$, we measured the PNC films’ linear absorption coefficient $\alpha_{\mathrm{film}}$ [the thickness measurement was performed by Atomic Force Microscopy (AFM) – Figure S6a], and compare it with the solution’s absorption coefficient at the same spectral region. The relative PNC concentrations in the films is therefore given by:

|  | $\chi_{\mathrm{film}}^{'}\equiv\frac{c_{\mathrm{film}}}{c_{0}}=\frac{\alpha_{\mathrm{film}}\left( 520 \mathrm{nm} \right)}{\alpha_{\mathrm{solution}}\left( 520 \mathrm{nm} \right)}.$ | (S24) |
| --- | --- | --- |

Here, $c_{0}$ is the standard concentration; and $c_{\mathrm{film}}$ is the concentration of the PNCs in the films. The PNC films’ absorption coefficient spectra are shown in Figure S6b. The calculated result is presented in Table S2.

The photon diffusion length due to photon recycling process ($L_{\mathrm{PR}}$) could be understood as the average Brownian motion of the photon in *x*- and *y*- directions before the photon escape from the system, that is:

|  | $L_{\mathrm{PR}}^{2}=\left\langle M_{\mathrm{film}} \right\rangle\left[ \left\langle x^{2} \right\rangle+\left\langle y^{2} \right\rangle\right]=\frac{2}{3}\left\langle M \right\rangle_{\mathrm{film}}\lambda_{\mathrm{film}}^{2}.$ | (S25) |
| --- | --- | --- |

Here, average number of PR occurrence in the PNC films $\left\langle M \right\rangle_{\mathrm{film}}$ could be calculated from the modified Eq. (S17) using the films’ absorbance at 2.62 eV (473 nm, CW pump photon energy for diffusion measurement):

|  | $\left\langle M \right\rangle_{\mathrm{film}}=6\xi_{2.62 \mathrm{eV}}^{2}\left( 1-\frac{A_{\mathrm{film}}e^{-A_{\mathrm{film}}}\left( 1+{A_{\mathrm{film}}}/2 \right)}{1-e^{-A_{\mathrm{film}}}} \right),$ | (S26) |
| --- | --- | --- |

where $\xi_{2.62 eV}\equiv\xi_{3.1 \mathrm{eV}}{A\left( 3.1 \mathrm{eV} \right)}/{A \left( 2.62 \mathrm{eV} \right)}$ is the ratio of absorption cross-section at PL and pump energy; and $A\left( \hbar\omega\right)$ is the film absorption at $\hbar\omega$. From the values of $\left\langle M \right\rangle_{\mathrm{film}}$, we could also predict their effective lifetimes:

|  | $\tau_{\mathrm{film}}=\tau_{0}\left( 1+\left\langle M \right\rangle_{\mathrm{film}} \right).$ | (S27) |
| --- | --- | --- |

The result corroborates our time-resolved PL measurement, and is also presented in Table S2. The large uncertainty in the prediction stems from the film roughness.

**Table S2 | Calculation for PNC films.** The parameters and calculation results for the photon recycling model in the case of PNC films are presented.

|  | **Hexyl** | **Octyl** | **Oleyl** |
| --- | --- | --- | --- |
| $A_{\mathrm{film}}$ at 473 nm (0.434 OD) | 1.848 | 2.236 | 4.202 |
| Thickness (μm) | 1.1 ± 0.1 | 0.64 ± 0.04 | 0.69 ± 0.05 |
| $\chi_{\mathrm{film}}^{'}$ | 320 ± 50 | 450 ± 20 | 840 ± 60 |
| $\lambda_{\mathrm{film}}$ (nm) | 1100 ± 300 | 830 ± 130 | 520 ± 140 |
| $\left\langle M \right\rangle_{\mathrm{film}}$ | 0.55 ± 0.15 | 0.48 ± 0.08 | 0.63 ± 0.16 |
| $\tau_{\mathrm{film}}$ (ns), predicted | 42 ± 12 | 31 ± 5 | 33 ± 9 |
| $\tau_{\mathrm{film}}$ (ns), experiment* | 33 ± 1 | 27 ± 1 | 30 ± 1 |
| *Measured at 3.1 eV pump with fluence corresponding to $\left\langle N \right\rangle$ ~ 0.6 exciton per NC (*i.e.*, identical condition with measurement in Fig. 2e, Main Text). | | | |

# Supplementary Note 7: Measurement on CdSe/ZnS core-shell QD film

As a validity check on the setup, we performed another measurement for commercial CdSe/ZnS core/shell QD system (EviDots ED-C11-TOL-0540, with diameter of ~2.4 nm and emission peak of ~535 nm, capped with long alkyl ligands). The absorption and PL spectra of such system are shown in Figure S8a. Due to overlapping region of the absorption and emission spectra, photon recycling process (PR) is still expected to occur. This is evident from our concentration-dependent measurement of the QD solution (Figure S8b), which is well described by our PR model. We fabricated a thin QD film for the diffusion measurement. The detailed fitting results and calculated parameters are presented in Table S3.

**Table S3 | Calculation for CdSe/ZnS samples.** The parameters and calculation results for the PR model in the case of CdSe/ZnS solution and film are presented.

| $\tau_{0}$ (ns) | 15.2 ± 0.1 |
| --- | --- |
| $\xi$ (400 nm) | 0.145 ± 0.005 |
| $A_{0}$ (0.434 OD) | 4.3 ± 0.7 |
| $\lambda_{0}$ (mm) | 1.6 ± 0.3 |
| $A_{\mathrm{film}}$ (0.434 OD) at 400 nm | 0.250 |
| $A_{\mathrm{film}}$ (0.434 OD) at 473 nm | 0.109 |
| $\xi$ (473 nm) | 0.33 ± 0.01 |
| $\alpha_{\mathrm{solution}}$ (cm^-1^) at 520 nm | 17.3 |
| $\alpha_{\mathrm{film}}$ (cm^-1^) at 520 nm | 1960 ± 100 |
| $\chi_{\mathrm{film}}^{'}$ | 110 ± 10 |
| $\left\langle M \right\rangle_{\mathrm{film}}$ | 0.0013 ± 0.0002 |
| $\lambda_{\mathrm{film}}$ (μm) | 14 ± 3 |
| $L_{\mathrm{PR}}$ (nm) | 400 ± 100 |

Based on these parameters, we estimated the contribution from PR to be $L_{\mathrm{PR}}$ = 400 ± 100 nm. In the earlier work by Tisdale’s group (*i.e.*, *J. Phys. Chem. C 2015, 119, 17, 9005–9015; Nano Lett. 2014, 14, 6, 3556–3562*), exciton hopping (EH) process in such QD film will be limited to several tens of nm and can be neglected (*i.e.*, beyond our spatial resolution). Thus, using our model, we estimated the total diffusion length in our CdSe/ZnS QD film to be $L_{D}=\sqrt{L_{\mathrm{EH}}^{2}+L_{\mathrm{PR}}^{2}}\approx L_{\mathrm{PR}}$ = 400 ± 100 nm, (*i.e.*, dominated by PR effect). Indeed, this is consistent with our PL imaging measurement (Figure S8c), where we obtained a diffusion length $L_{D}$ = 330 ± 70 nm in the thin QD film. Such result validates the accuracy and consistency of both our model and imaging technique.
